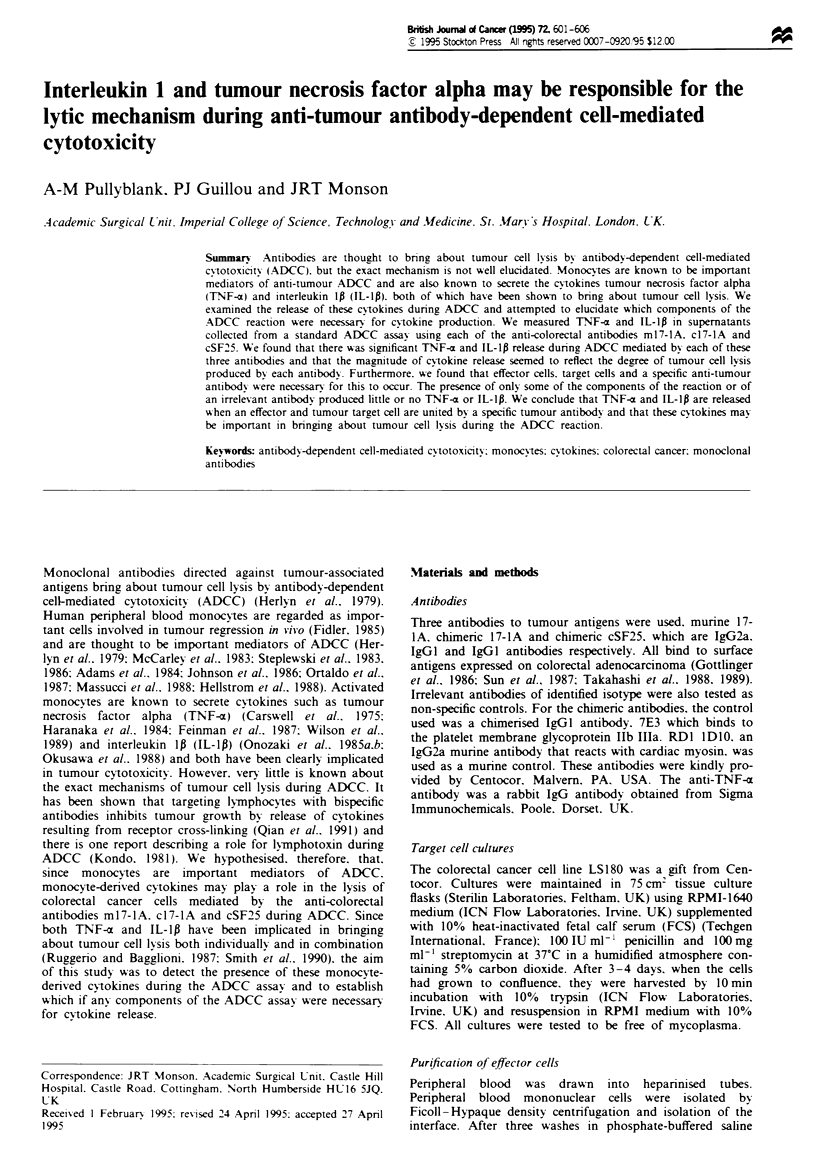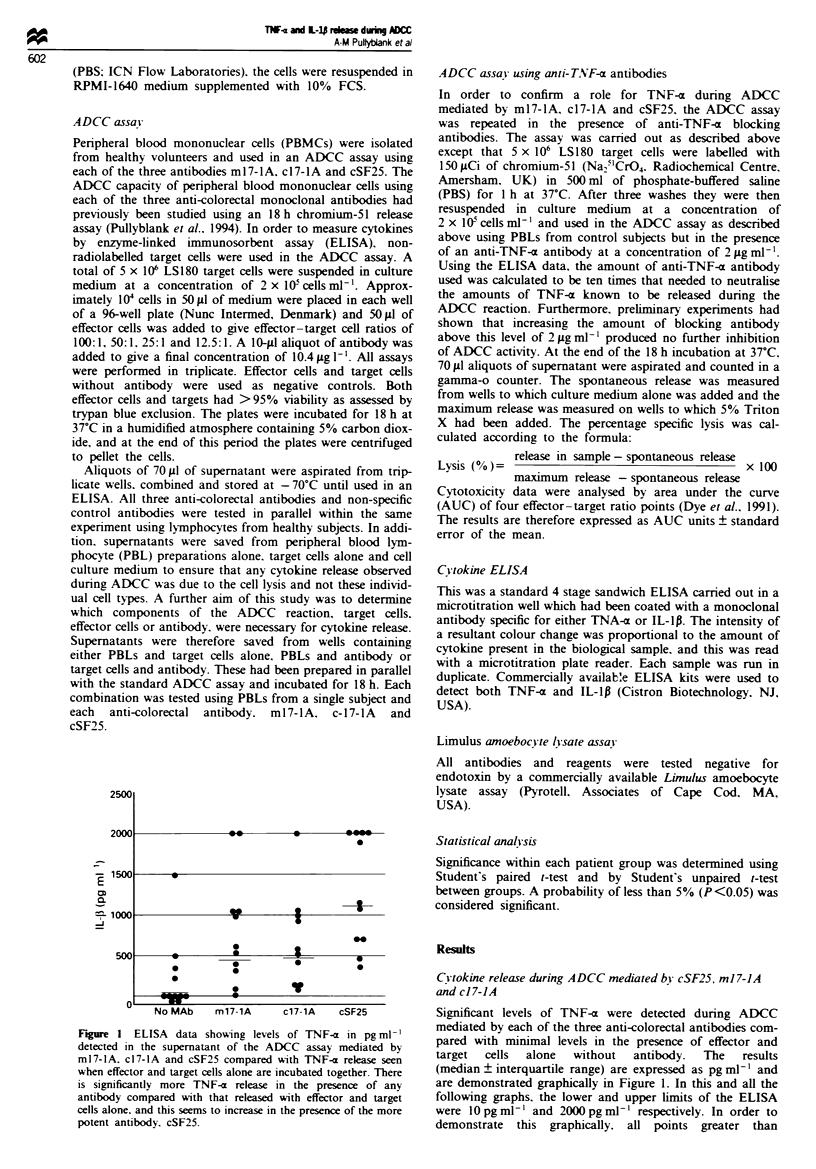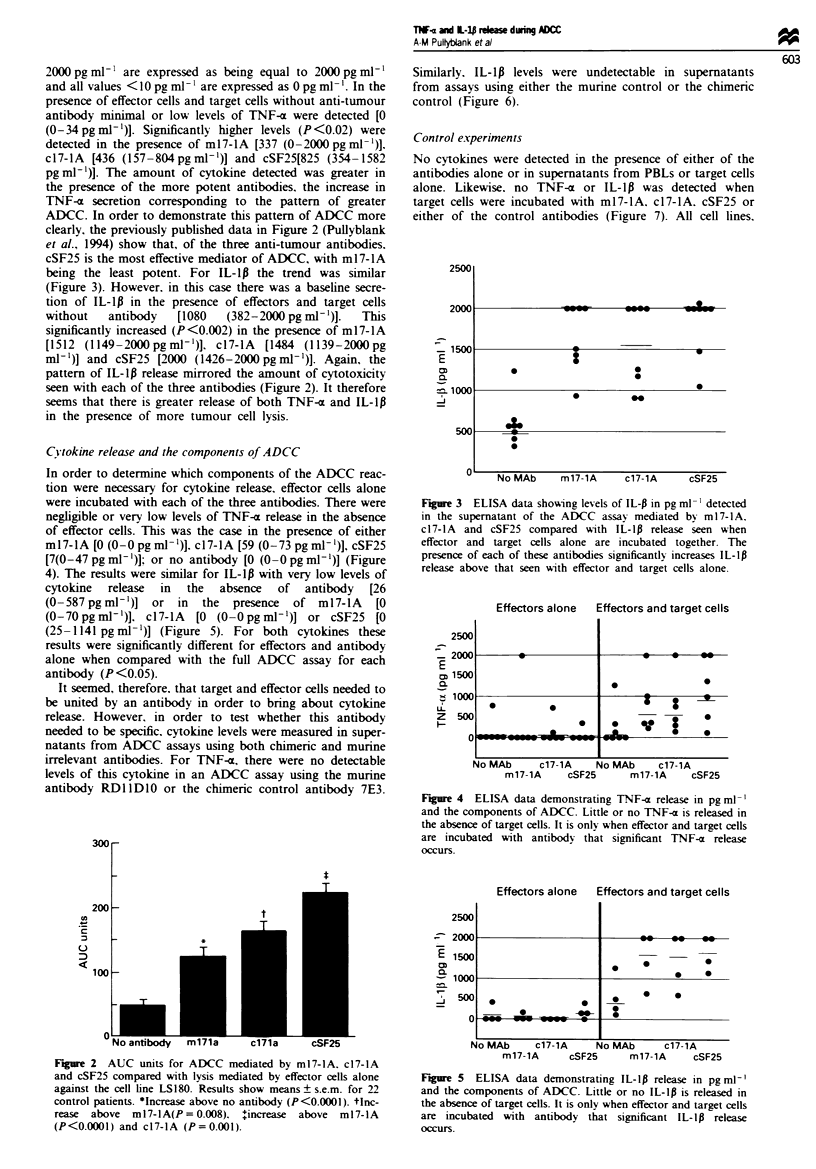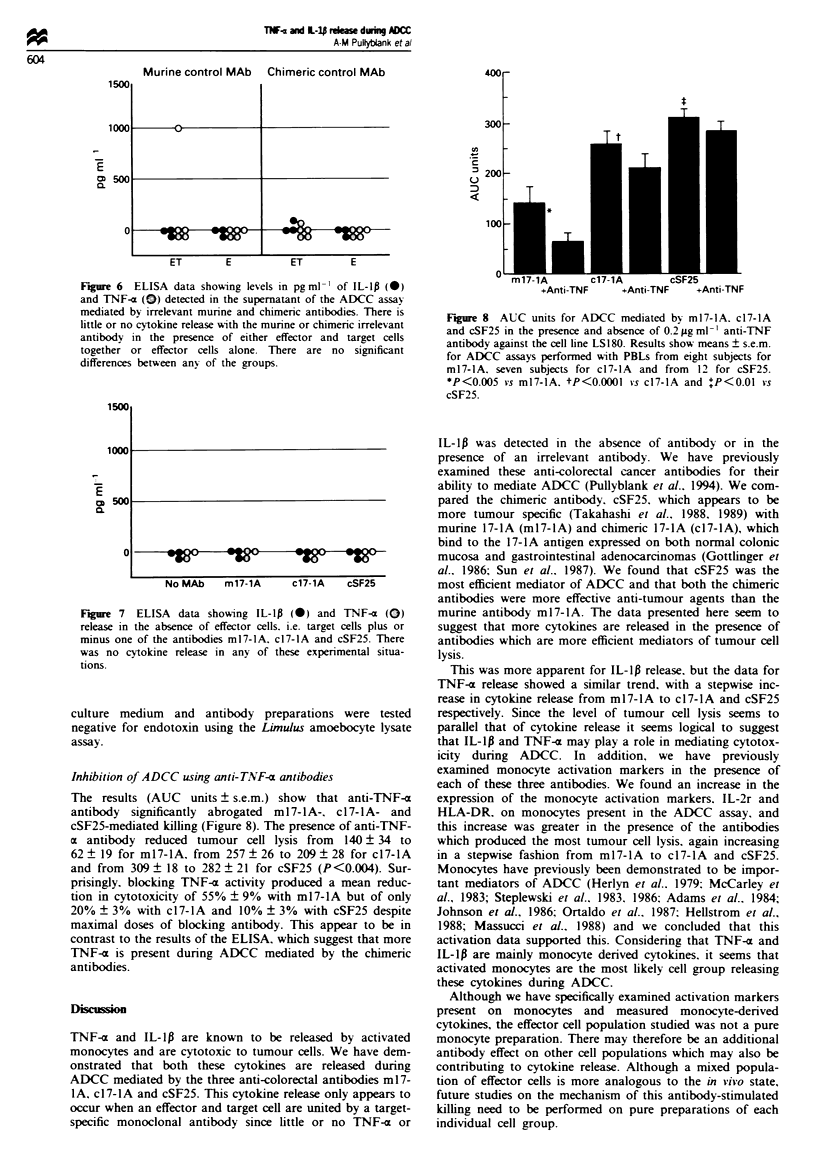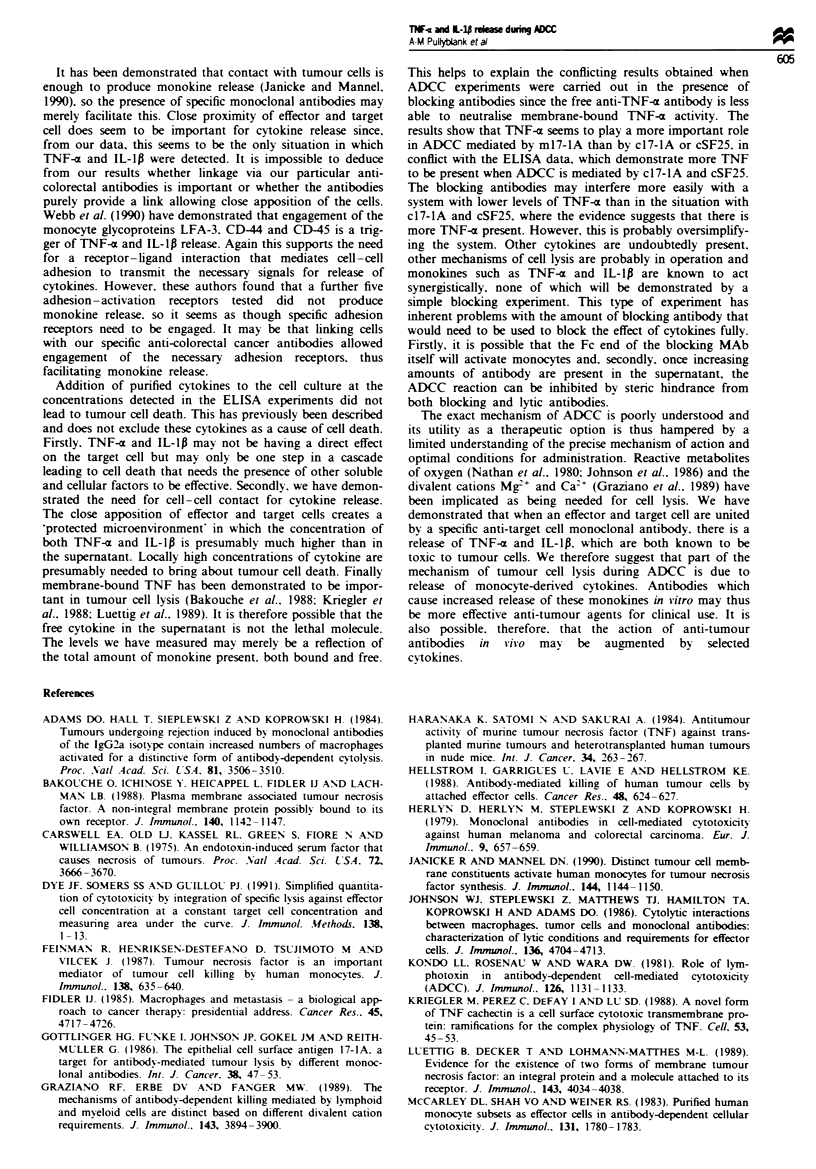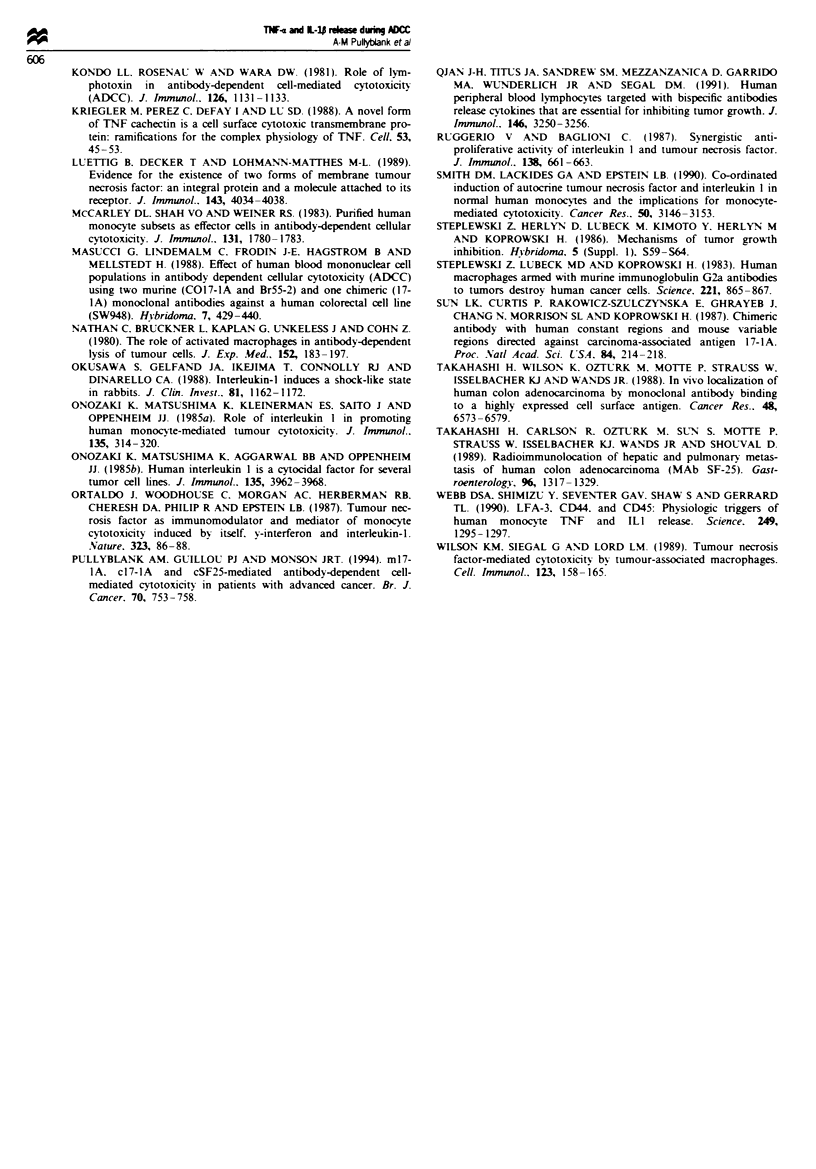# Interleukin 1 and tumour necrosis factor alpha may be responsible for the lytic mechanism during anti-tumour antibody-dependent cell-mediated cytotoxicity.

**DOI:** 10.1038/bjc.1995.380

**Published:** 1995-09

**Authors:** A. M. Pullyblank, P. J. Guillou, J. R. Monson

**Affiliations:** Academic Surgical Unit, Imperial College of Science, Technology and Medicine, St. Mary's Hospital, London, UK.

## Abstract

Antibodies are thought to bring about tumour cell lysis by antibody-dependent cell-mediated cytotoxicity (ADCC), but the exact mechanism is not well elucidated. Monocytes are known to be important mediators of anti-tumour ADCC and are also known to secrete the cytokines tumour necrosis factor alpha (TNF-alpha) and interleukin 1 beta (IL-1 beta), both of which have been shown to bring about tumour cell lysis. We examined the release of these cytokines during ADCC and attempted to elucidate which components of the ADCC reaction were necessary for cytokine production. We measured TNF-alpha and IL-1 beta in supernatants collected from a standard ADCC assay using each of the anti-colorectal antibodies m17-1A, c17-1A and cSF25. We found that there was significant TNF-alpha and IL-1 beta release during ADCC mediated by each of these three antibodies and that the magnitude of cytokine release seemed to reflect the degree of tumour cell lysis produced by each antibody. Furthermore, we found that effector cells, target cells and a specific anti-tumour antibody were necessary for this to occur. The presence of only some of the components of the reaction or of an irrelevant antibody produced little or no TNF-alpha or IL-1 beta. We conclude that TNF-alpha and IL-1 beta are released when an effector and tumour target cell are united by a specific tumour antibody and that these cytokines may be important in bringing about tumour cell lysis during the ADCC reaction.


British Journal of Cancer (1995) 72, 601-606

© 1995 Stockton Press All rights reserved 0007-0920/95 $12.00

### Interleukin 1 and tumour necrosis factor alpha may be responsible for the lytic mechanism during anti-tumour antibody-dependent cell-mediated cytotoxicity

A-M Pullyblank, PJ Guillou and JRT Monson

*Academic Surgical Unit, Imperial College of Science, Technology and Medicine, St. Mary's Hospital, London, UK.*

**Summary** Antibodies are thought to bring about tumour cell lysis by antibody-dependent cell-mediated cytotoxicity (ADCC), but the exact mechanism is not well elucidated. Monocytes are known to be important mediators of anti-tumour ADCC and are also known to secrete the cytokines tumour necrosis factor alpha (TNF-α) and interleukin 1β (IL-1β), both of which have been shown to bring about tumour cell lysis. We examined the release of these cytokines during ADCC and attempted to elucidate which components of the ADCC reaction were necessary for cytokine production. We measured TNF-α and IL-1β in supernatants collected from a standard ADCC assay using each of the anti-colorectal antibodies m17-1A, c17-1A and cSF25. We found that there was significant TNF-α and IL-1β release during ADCC mediated by each of these three antibodies and that the magnitude of cytokine release seemed to reflect the degree of tumour cell lysis produced by each antibody. Furthermore, we found that effector cells, target cells and a specific anti-tumour antibody were necessary for this to occur. The presence of only some of the components of the reaction or of an irrelevant antibody produced little or no TNF-α or IL-1β. We conclude that TNF-α and IL-1β are released when an effector and tumour target cell are united by a specific tumour antibody and that these cytokines may be important in bringing about tumour cell lysis during the ADCC reaction.

**Keywords:** antibody-dependent cell-mediated cytotoxicity; monocytes; cytokines; colorectal cancer; monoclonal antibodies

Monoclonal antibodies directed against tumour-associated antigens bring about tumour cell lysis by antibody-dependent cell-mediated cytotoxicity (ADCC) (Herlyn *et al.*, 1979). Human peripheral blood monocytes are regarded as important cells involved in tumour regression *in vivo* (Fidler, 1985) and are thought to be important mediators of ADCC (Herlyn *et al.*, 1979; McCarley *et al.*, 1983; Steplewski *et al.*, 1983, 1986; Adams *et al.*, 1984; Johnson *et al.*, 1986; Ortaldo *et al.*, 1987; Massucci *et al.*, 1988; Hellstrom *et al.*, 1988). Activated monocytes are known to secrete cytokines such as tumour necrosis factor alpha (TNF-α) (Carswell *et al.*, 1975; Haranaka *et al.*, 1984; Feinman *et al.*, 1987; Wilson *et al.*, 1989) and interleukin 1β (IL-1β) (Onozaki *et al.*, 1985*a,b*; Okusawa *et al.*, 1988) and both have been clearly implicated in tumour cytotoxicity. However, very little is known about the exact mechanisms of tumour cell lysis during ADCC. It has been shown that targeting lymphocytes with bispecific antibodies inhibits tumour growth by release of cytokines resulting from receptor cross-linking (Qian *et al.*, 1991) and there is one report describing a role for lymphotoxin during ADCC (Kondo, 1981). We hypothesised, therefore, that, since monocytes are important mediators of ADCC, monocyte-derived cytokines may play a role in the lysis of colorectal cancer cells mediated by the anti-colorectal antibodies m17-1A, c17-1A and cSF25 during ADCC. Since both TNF-α and IL-1β have been implicated in bringing about tumour cell lysis both individually and in combination (Ruggerio and Bagglioni, 1987; Smith *et al.*, 1990), the aim of this study was to detect the presence of these monocyte-derived cytokines during the ADCC assay and to establish which if any components of the ADCC assay were necessary for cytokine release.

Correspondence: JRT Monson, Academic Surgical Unit, Castle Hill Hospital, Castle Road, Cottingham, North Humberside HU16 5JQ, UK
Received 1 February 1995; revised 24 April 1995; accepted 27 April 1995

### Materials and methods

### *Antibodies*

Three antibodies to tumour antigens were used, murine 17-1A, chimeric 17-1A and chimeric cSF25, which are IgG2a, IgG1 and IgG1 antibodies respectively. All bind to surface antigens expressed on colorectal adenocarcinoma (Gottlinger *et al.*, 1986; Sun *et al.*, 1987; Takahashi *et al.*, 1988, 1989). Irrelevant antibodies of identified isotype were also tested as non-specific controls. For the chimeric antibodies, the control used was a chimerised IgG1 antibody, 7E3 which binds to the platelet membrane glycoprotein IIb IIIa. RD1 1D10, an IgG2a murine antibody that reacts with cardiac myosin, was used as a murine control. These antibodies were kindly provided by Centocor, Malvern, PA, USA. The anti-TNF-α antibody was a rabbit IgG antibody obtained from Sigma Immunochemicals, Poole, Dorset, UK.

### *Target cell cultures*

The colorectal cancer cell line LS180 was a gift from Centocor. Cultures were maintained in 75 $cm^2$ tissue culture flasks (Sterilin Laboratories, Feltham, UK) using RPMI-1640 medium (ICN Flow Laboratories, Irvine, UK) supplemented with 10% heat-inactivated fetal calf serum (FCS) (Techgen International, France); 100 IU $ml^{-1}$ penicillin and 100 mg $ml^{-1}$ streptomycin at 37°C in a humidified atmosphere containing 5% carbon dioxide. After 3-4 days, when the cells had grown to confluence, they were harvested by 10 min incubation with 10% trypsin (ICN Flow Laboratories, Irvine, UK) and resuspension in RPMI medium with 10% FCS. All cultures were tested to be free of mycoplasma.

### *Purification of effector cells*

Peripheral blood was drawn into heparinised tubes. Peripheral blood mononuclear cells were isolated by Ficoll-Hypaque density centrifugation and isolation of the interface. After three washes in phosphate-buffered saline

(PBS; ICN Flow Laboratories), the cells were resuspended in RPMI-1640 medium supplemented with 10% FCS.

*ADCC assay*

Peripheral blood mononuclear cells (PBMCs) were isolated from healthy volunteers and used in an ADCC assay using each of the three antibodies m17-1A, c17-1A and cSF25. The ADCC capacity of peripheral blood mononuclear cells using each of the three anti-colorectal monoclonal antibodies had previously been studied using an 18 h chromium-51 release assay (Pullyblank *et al.*, 1994). In order to measure cytokines by enzyme-linked immunosorbent assay (ELISA), non-radiolabelled target cells were used in the ADCC assay. A total of $5 \times 10^6$ LS180 target cells were suspended in culture medium at a concentration of $2 \times 10^5$ cells $ml^{-1}$. Approximately $10^4$ cells in 50 μl of medium were placed in each well of a 96-well plate (Nunc Intermed, Denmark) and 50 μl of effector cells was added to give effector–target cell ratios of 100:1, 50:1, 25:1 and 12.5:1. A 10-μl aliquot of antibody was added to give a final concentration of 10.4 μg $l^{-1}$. All assays were performed in triplicate. Effector cells and target cells without antibody were used as negative controls. Both effector cells and targets had >95% viability as assessed by trypan blue exclusion. The plates were incubated for 18 h at 37°C in a humidified atmosphere containing 5% carbon dioxide, and at the end of this period the plates were centrifuged to pellet the cells.

Aliquots of 70 μl of supernatant were aspirated from triplicate wells, combined and stored at −70°C until used in an ELISA. All three anti-colorectal antibodies and non-specific control antibodies were tested in parallel within the same experiment using lymphocytes from healthy subjects. In addition, supernatants were saved from peripheral blood lymphocyte (PBL) preparations alone, target cells alone and cell culture medium to ensure that any cytokine release observed during ADCC was due to the cell lysis and not these individual cell types. A further aim of this study was to determine which components of the ADCC reaction, target cells, effector cells or antibody, were necessary for cytokine release. Supernatants were therefore saved from wells containing either PBLs and target cells alone, PBLs and antibody or target cells and antibody. These had been prepared in parallel with the standard ADCC assay and incubated for 18 h. Each combination was tested using PBLs from a single subject and each anti-colorectal antibody, m17-1A, c-17-1A and cSF25.

**Figure 1** ELISA data showing levels of TNF-α in pg $ml^{-1}$ detected in the supernatant of the ADCC assay mediated by m17-1A, c17-1A and cSF25 compared with TNF-α release seen when effector and target cells alone are incubated together. There is significantly more TNF-α release in the presence of any antibody compared with that released with effector and target cells alone, and this seems to increase in the presence of the more potent antibody, cSF25.

*ADCC assay using anti-TNF-α antibodies*

In order to confirm a role for TNF-α during ADCC mediated by m17-1A, c17-1A and cSF25, the ADCC assay was repeated in the presence of anti-TNF-α blocking antibodies. The assay was carried out as described above except that $5 \times 10^6$ LS180 target cells were labelled with 150 μCi of chromium-51 ($Na_2{}^{51}CrO_4$, Radiochemical Centre, Amersham, UK) in 500 ml of phosphate-buffered saline (PBS) for 1 h at 37°C. After three washes they were then resuspended in culture medium at a concentration of $2 \times 10^5$ cells $ml^{-1}$ and used in the ADCC assay as described above using PBLs from control subjects but in the presence of an anti-TNF-α antibody at a concentration of 2 μg $ml^{-1}$. Using the ELISA data, the amount of anti-TNF-α antibody used was calculated to be ten times that needed to neutralise the amounts of TNF-α known to be released during the ADCC reaction. Furthermore, preliminary experiments had shown that increasing the amount of blocking antibody above this level of 2 μg $ml^{-1}$ produced no further inhibition of ADCC activity. At the end of the 18 h incubation at 37°C, 70 μl aliquots of supernatant were aspirated and counted in a gamma-o counter. The spontaneous release was measured from wells to which culture medium alone was added and the maximum release was measured on wells to which 5% Triton X had been added. The percentage specific lysis was calculated according to the formula:

$$\text{Lysis (\%)} = \frac{\text{release in sample} - \text{spontaneous release}}{\text{maximum release} - \text{spontaneous release}} \times 100$$

Cytotoxicity data were analysed by area under the curve (AUC) of four effector–target ratio points (Dye *et al.*, 1991). The results are therefore expressed as AUC units ± standard error of the mean.

*Cytokine ELISA*

This was a standard 4 stage sandwich ELISA carried out in a microtitration well which had been coated with a monoclonal antibody specific for either TNA-α or IL-1β. The intensity of a resultant colour change was proportional to the amount of cytokine present in the biological sample, and this was read with a microtitration plate reader. Each sample was run in duplicate. Commercially available ELISA kits were used to detect both TNF-α and IL-1β (Cistron Biotechnology, NJ, USA).

Limulus *amoebocyte lysate assay*

All antibodies and reagents were tested negative for endotoxin by a commercially available *Limulus* amoebocyte lysate assay (Pyrotell, Associates of Cape Cod, MA, USA).

*Statistical analysis*

Significance within each patient group was determined using Student's paired *t*-test and by Student's unpaired *t*-test between groups. A probability of less than 5% ($P<0.05$) was considered significant.

### Results

*Cytokine release during ADCC mediated by cSF25, m17-1A and c17-1A*

Significant levels of TNF-α were detected during ADCC mediated by each of the three anti-colorectal antibodies compared with minimal levels in the presence of effector and target cells alone without antibody. The results (median ± interquartile range) are expressed as pg $ml^{-1}$ and are demonstrated graphically in Figure 1. In this and all the following graphs, the lower and upper limits of the ELISA were 10 pg $ml^{-1}$ and 2000 pg $ml^{-1}$ respectively. In order to demonstrate this graphically, all points greater than

2000 pg ml$^{-1}$ are expressed as being equal to 2000 pg ml$^{-1}$ and all values <10 pg ml$^{-1}$ are expressed as 0 pg ml$^{-1}$. In the presence of effector cells and target cells without anti-tumour antibody minimal or low levels of TNF-α were detected [0 (0–34 pg ml$^{-1}$)]. Significantly higher levels ($P$<0.02) were detected in the presence of m17-1A [337 (0–2000 pg ml$^{-1}$)], c17-1A [436 (157–804 pg ml$^{-1}$)] and cSF25[825 (354–1582 pg ml$^{-1}$)]. The amount of cytokine detected was greater in the presence of the more potent antibodies, the increase in TNF-α secretion corresponding to the pattern of greater ADCC. In order to demonstrate this pattern of ADCC more clearly, the previously published data in Figure 2 (Pullyblank *et al.*, 1994) show that, of the three anti-tumour antibodies, cSF25 is the most effective mediator of ADCC, with m17-1A being the least potent. For IL-1β the trend was similar (Figure 3). However, in this case there was a baseline secretion of IL-1β in the presence of effectors and target cells without antibody [1080 (382–2000 pg ml$^{-1}$)]. This significantly increased ($P$<0.002) in the presence of m17-1A [1512 (1149–2000 pg ml$^{-1}$)], c17-1A [1484 (1139–2000 pg ml$^{-1}$)] and cSF25 [2000 (1426–2000 pg ml$^{-1}$)]. Again, the pattern of IL-1β release mirrored the amount of cytotoxicity seen with each of the three antibodies (Figure 2). It therefore seems that there is greater release of both TNF-α and IL-1β in the presence of more tumour cell lysis.

### *Cytokine release and the components of ADCC*

In order to determine which components of the ADCC reaction were necessary for cytokine release, effector cells alone were incubated with each of the three antibodies. There were negligible or very low levels of TNF-α release in the absence of effector cells. This was the case in the presence of either m17-1A [0 (0–0 pg ml$^{-1}$)], c17-1A [59 (0–73 pg ml$^{-1}$)], cSF25 [7(0–47 pg ml$^{-1}$)]; or no antibody [0 (0–0 pg ml$^{-1}$)] (Figure 4). The results were similar for IL-1β with very low levels of cytokine release in the absence of antibody [26 (0–587 pg ml$^{-1}$)] or in the presence of m17-1A [0 (0–70 pg ml$^{-1}$)], c17-1A [0 (0–0 pg ml$^{-1}$)] or cSF25 [0 (25–1141 pg ml$^{-1}$)] (Figure 5). For both cytokines these results were significantly different for effectors and antibody alone when compared with the full ADCC assay for each antibody ($P$<0.05).

It seemed, therefore, that target and effector cells needed to be united by an antibody in order to bring about cytokine release. However, in order to test whether this antibody needed to be specific, cytokine levels were measured in supernatants from ADCC assays using both chimeric and murine irrelevant antibodies. For TNF-α, there were no detectable levels of this cytokine in an ADCC assay using the murine antibody RD11D10 or the chimeric control antibody 7E3.

**Figure 2** AUC units for ADCC mediated by m17-1A, c17-1A and cSF25 compared with lysis mediated by effector cells alone against the cell line LS180. Results show means ± s.e.m. for 22 control patients. *Increase above no antibody ($P$<0.0001). †Increase above m17-1A($P$ = 0.008). ‡increase above m17-1A ($P$<0.0001) and c17-1A ($P$ = 0.001).

Similarly, IL-1β levels were undetectable in supernatants from assays using either the murine control or the chimeric control (Figure 6).

### *Control experiments*

No cytokines were detected in the presence of either of the antibodies alone or in supernatants from PBLs or target cells alone. Likewise, no TNF-α or IL-1β was detected when target cells were incubated with m17-1A, c17-1A, cSF25 or either of the control antibodies (Figure 7). All cell lines,

**Figure 3** ELISA data showing levels of IL-β in pg ml$^{-1}$ detected in the supernatant of the ADCC assay mediated by m17-1A, c17-1A and cSF25 compared with IL-1β release seen when effector and target cells alone are incubated together. The presence of each of these antibodies significantly increases IL-1β release above that seen with effector and target cells alone.

**Figure 4** ELISA data demonstrating TNF-α release in pg ml$^{-1}$ and the components of ADCC. Little or no TNF-α is released in the absence of target cells. It is only when effector and target cells are incubated with antibody that significant TNF-α release occurs.

**Figure 5** ELISA data demonstrating IL-1β release in pg ml$^{-1}$ and the components of ADCC. Little or no IL-1β is released in the absence of target cells. It is only when effector and target cells are incubated with antibody that significant IL-1β release occurs.

**Figure 6** ELISA data showing levels in pg $ml^{-1}$ of IL-1β (●) and TNF-α (◎) detected in the supernatant of the ADCC assay mediated by irrelevant murine and chimeric antibodies. There is little or no cytokine release with the murine or chimeric irrelevant antibody in the presence of either effector and target cells together or effector cells alone. There are no significant differences between any of the groups.

**Figure 7** ELISA data showing IL-1β (●) and TNF-α (◎) release in the absence of effector cells, i.e. target cells plus or minus one of the antibodies m17-1A, c17-1A and cSF25. There was no cytokine release in any of these experimental situations.

**Figure 8** AUC units for ADCC mediated by m17-1A, c17-1A and cSF25 in the presence and absence of 0.2 μg $ml^{-1}$ anti-TNF antibody against the cell line LS180. Results show means ± s.e.m. for ADCC assays performed with PBLs from eight subjects for m17-1A, seven subjects for c17-1A and from 12 for cSF25. $^{*}P<0.005$ *vs* m17-1A, $^{\dagger}P<0.0001$ *vs* c17-1A and $^{\ddagger}P<0.01$ *vs* cSF25.

culture medium and antibody preparations were tested negative for endotoxin using the *Limulus* amoebocyte lysate assay.

*Inhibition of ADCC using anti-TNF-α antibodies*

The results (AUC units ± s.e.m.) show that anti-TNF-α antibody significantly abrogated m17-1A-, c17-1A- and cSF25-mediated killing (Figure 8). The presence of anti-TNF-α antibody reduced tumour cell lysis from 140 ± 34 to 62 ± 19 for m17-1A, from 257 ± 26 to 209 ± 28 for c17-1A and from 309 ± 18 to 282 ± 21 for cSF25 ($P<0.004$). Surprisingly, blocking TNF-α activity produced a mean reduction in cytotoxicity of 55% ± 9% with m17-1A but of only 20% ± 3% with c17-1A and 10% ± 3% with cSF25 despite maximal doses of blocking antibody. This appear to be in contrast to the results of the ELISA, which suggest that more TNF-α is present during ADCC mediated by the chimeric antibodies.

### Discussion

TNF-α and IL-1β are known to be released by activated monocytes and are cytotoxic to tumour cells. We have demonstrated that both these cytokines are released during ADCC mediated by the three anti-colorectal antibodies m17-1A, c17-1A and cSF25. This cytokine release only appears to occur when an effector and target cell are united by a target-specific monoclonal antibody since little or no TNF-α or IL-1β was detected in the absence of antibody or in the presence of an irrelevant antibody. We have previously examined these anti-colorectal cancer antibodies for their ability to mediate ADCC (Pullyblank *et al*., 1994). We compared the chimeric antibody, cSF25, which appears to be more tumour specific (Takahashi *et al*., 1988, 1989) with murine 17-1A (m17-1A) and chimeric 17-1A (c17-1A), which bind to the 17-1A antigen expressed on both normal colonic mucosa and gastrointestinal adenocarcinomas (Gottlinger *et al*., 1986; Sun *et al*., 1987). We found that cSF25 was the most efficient mediator of ADCC and that both the chimeric antibodies were more effective anti-tumour agents than the murine antibody m17-1A. The data presented here seem to suggest that more cytokines are released in the presence of antibodies which are more efficient mediators of tumour cell lysis.

This was more apparent for IL-1β release, but the data for TNF-α release showed a similar trend, with a stepwise increase in cytokine release from m17-1A to c17-1A and cSF25 respectively. Since the level of tumour cell lysis seems to parallel that of cytokine release it seems logical to suggest that IL-1β and TNF-α may play a role in mediating cytotoxicity during ADCC. In addition, we have previously examined monocyte activation markers in the presence of each of these three antibodies. We found an increase in the expression of the monocyte activation markers, IL-2r and HLA-DR, on monocytes present in the ADCC assay, and this increase was greater in the presence of the antibodies which produced the most tumour cell lysis, again increasing in a stepwise fashion from m17-1A to c17-1A and cSF25. Monocytes have previously been demonstrated to be important mediators of ADCC (Herlyn *et al*., 1979; McCarley *et al*., 1983; Steplewski *et al*., 1983, 1986; Adams *et al*., 1984; Johnson *et al*., 1986; Ortaldo *et al*., 1987; Hellstrom *et al*., 1988; Massucci *et al*., 1988) and we concluded that this activation data supported this. Considering that TNF-α and IL-1β are mainly monocyte derived cytokines, it seems that activated monocytes are the most likely cell group releasing these cytokines during ADCC.

Although we have specifically examined activation markers present on monocytes and measured monocyte-derived cytokines, the effector cell population studied was not a pure monocyte preparation. There may therefore be an additional antibody effect on other cell populations which may also be contributing to cytokine release. Although a mixed population of effector cells is more analogous to the *in vivo* state, future studies on the mechanism of this antibody-stimulated killing need to be performed on pure preparations of each individual cell group.

It has been demonstrated that contact with tumour cells is enough to produce monokine release (Janicke and Mannel, 1990), so the presence of specific monoclonal antibodies may merely facilitate this. Close proximity of effector and target cell does seem to be important for cytokine release since, from our data, this seems to be the only situation in which TNF-α and IL-1β were detected. It is impossible to deduce from our results whether linkage via our particular anti-colorectal antibodies is important or whether the antibodies purely provide a link allowing close apposition of the cells. Webb *et al.* (1990) have demonstrated that engagement of the monocyte glycoproteins LFA-3, CD-44 and CD-45 is a trigger of TNF-α and IL-1β release. Again this supports the need for a receptor–ligand interaction that mediates cell–cell adhesion to transmit the necessary signals for release of cytokines. However, these authors found that a further five adhesion–activation receptors tested did not produce monokine release, so it seems as though specific adhesion receptors need to be engaged. It may be that linking cells with our specific anti-colorectal cancer antibodies allowed engagement of the necessary adhesion receptors, thus facilitating monokine release.

Addition of purified cytokines to the cell culture at the concentrations detected in the ELISA experiments did not lead to tumour cell death. This has previously been described and does not exclude these cytokines as a cause of cell death. Firstly, TNF-α and IL-1β may not be having a direct effect on the target cell but may only be one step in a cascade leading to cell death that needs the presence of other soluble and cellular factors to be effective. Secondly, we have demonstrated the need for cell–cell contact for cytokine release. The close apposition of effector and target cells creates a 'protected microenvironment' in which the concentration of both TNF-α and IL-1β is presumably much higher than in the supernatant. Locally high concentrations of cytokine are presumably needed to bring about tumour cell death. Finally membrane-bound TNF has been demonstrated to be important in tumour cell lysis (Bakouche *et al.*, 1988; Kriegler *et al.*, 1988; Luettig *et al.*, 1989). It is therefore possible that the free cytokine in the supernatant is not the lethal molecule. The levels we have measured may merely be a reflection of the total amount of monokine present, both bound and free. This helps to explain the conflicting results obtained when ADCC experiments were carried out in the presence of blocking antibodies since the free anti-TNF-α antibody is less able to neutralise membrane-bound TNF-α activity. The results show that TNF-α seems to play a more important role in ADCC mediated by m17-1A than by c17-1A or cSF25, in conflict with the ELISA data, which demonstrate more TNF to be present when ADCC is mediated by c17-1A and cSF25. The blocking antibodies may interfere more easily with a system with lower levels of TNF-α than in the situation with c17-1A and cSF25, where the evidence suggests that there is more TNF-α present. However, this is probably oversimplifying the system. Other cytokines are undoubtedly present, other mechanisms of cell lysis are probably in operation and monokines such as TNF-α and IL-1β are known to act synergistically, none of which will be demonstrated by a simple blocking experiment. This type of experiment has inherent problems with the amount of blocking antibody that would need to be used to block the effect of cytokines fully. Firstly, it is possible that the Fc end of the blocking MAb itself will activate monocytes and, secondly, once increasing amounts of antibody are present in the supernatant, the ADCC reaction can be inhibited by steric hindrance from both blocking and lytic antibodies.

The exact mechanism of ADCC is poorly understood and its utility as a therapeutic option is thus hampered by a limited understanding of the precise mechanism of action and optimal conditions for administration. Reactive metabolites of oxygen (Nathan *et al.*, 1980; Johnson *et al.*, 1986) and the divalent cations $Mg^{2+}$ and $Ca^{2+}$ (Graziano *et al.*, 1989) have been implicated as being needed for cell lysis. We have demonstrated that when an effector and target cell are united by a specific anti-target cell monoclonal antibody, there is a release of TNF-α and IL-1β, which are both known to be toxic to tumour cells. We therefore suggest that part of the mechanism of tumour cell lysis during ADCC is due to release of monocyte-derived cytokines. Antibodies which cause increased release of these monokines *in vitro* may thus be more effective anti-tumour agents for clinical use. It is also possible, therefore, that the action of anti-tumour antibodies *in vivo* may be augmented by selected cytokines.

### References

ADAMS DO, HALL T, SIEPLEWSKI Z AND KOPROWSKI H. (1984). Tumours undergoing rejection induced by monoclonal antibodies of the IgG2a isotype contain increased numbers of macrophages activated for a distinctive form of antibody-dependent cytolysis. *Proc. Natl Acad. Sci. USA*, **81**, 3506–3510.

BAKOUCHE O, ICHINOSE Y, HEICAPPEL L, FIDLER IJ AND LACHMAN LB. (1988). Plasma membrane associated tumour necrosis factor. A non-integral membrane protein possibly bound to its own receptor. *J. Immunol.*, **140**, 1142–1147.

CARSWELL EA, OLD LJ, KASSEL RL, GREEN S, FIORE N AND WILLIAMSON B. (1975). An endotoxin-induced serum factor that causes necrosis of tumours. *Proc. Natl Acad. Sci. USA*, **72**, 3666–3670.

DYE JF, SOMERS SS AND GUILLOU PJ. (1991). Simplified quantitation of cytotoxicity by integration of specific lysis against effector cell concentration at a constant target cell concentration and measuring area under the curve. *J. Immunol. Methods*, **138**, 1–13.

FEINMAN R, HENRIKSEN-DESTEFANO D, TSUJIMOTO M AND VILCEK J. (1987). Tumour necrosis factor is an important mediator of tumour cell killing by human monocytes. *J. Immunol.*, **138**, 635–640.

FIDLER IJ. (1985). Macrophages and metastasis – a biological approach to cancer therapy: presidential address. *Cancer Res.*, **45**, 4717–4726.

GOTTLINGER HG, FUNKE I, JOHNSON JP, GOKEL JM AND REITHMULLER G. (1986). The epithelial cell surface antigen 17-1A, a target for antibody-mediated tumour lysis by different monoclonal antibodies. *Int. J. Cancer*, **38**, 47–53.

GRAZIANO RF, ERBE DV AND FANGER MW. (1989). The mechanisms of antibody-dependent killing mediated by lymphoid and myeloid cells are distinct based on different divalent cation requirements. *J. Immunol.*, **143**, 3894–3900.

HARANAKA K, SATOMI N AND SAKURAI A. (1984). Antitumour activity of murine tumour necrosis factor (TNF) against transplanted murine tumours and heterotransplanted human tumours in nude mice. *Int. J. Cancer*, **34**, 263–267.

HELLSTROM I, GARRIGUES U, LAVIE E AND HELLSTROM KE. (1988). Antibody-mediated killing of human tumour cells by attached effector cells. *Cancer Res.*, **48**, 624–627.

HERLYN D, HERLYN M, STEPLEWSKI Z AND KOPROWSKI H. (1979). Monoclonal antibodies in cell-mediated cytotoxicity against human melanoma and colorectal carcinoma. *Eur. J. Immunol.*, **9**, 657–659.

JANICKE R AND MANNEL DN. (1990). Distinct tumour cell membrane constituents activate human monocytes for tumour necrosis factor synthesis. *J. Immunol.*, **144**, 1144–1150.

JOHNSON WJ, STEPLEWSKI Z, MATTHEWS TJ, HAMILTON TA, KOPROWSKI H AND ADAMS DO. (1986). Cytolytic interactions between macrophages, tumor cells and monoclonal antibodies: characterization of lytic conditions and requirements for effector cells. *J. Immunol.*, **136**, 4704–4713.

KONDO LL, ROSENAU W AND WARA DW. (1981). Role of lymphotoxin in antibody-dependent cell-mediated cytotoxicity (ADCC). *J. Immunol.*, **126**, 1131–1133.

KRIEGLER M, PEREZ C, DEFAY I AND LU SD. (1988). A novel form of TNF cachectin is a cell surface cytotoxic transmembrane protein: ramifications for the complex physiology of TNF. *Cell*, **53**, 45–53.

LUETTIG B, DECKER T AND LOHMANN-MATTHES M-L. (1989). Evidence for the existence of two forms of membrane tumour necrosis factor: an integral protein and a molecule attached to its receptor. *J. Immunol.*, **143**, 4034–4038.

MCCARLEY DL, SHAH VO AND WEINER RS. (1983). Purified human monocyte subsets as effector cells in antibody-dependent cellular cytotoxicity. *J. Immunol.*, **131**, 1780–1783.

KONDO LL, ROSENAU W AND WARA DW. (1981). Role of lymphotoxin in antibody-dependent cell-mediated cytotoxicity (ADCC). *J. Immunol.*, **126,** 1131–1133.

KRIEGLER M, PEREZ C, DEFAY I AND LU SD. (1988). A novel form of TNF cachectin is a cell surface cytotoxic transmembrane protein: ramifications for the complex physiology of TNF. *Cell*, **53,** 45–53.

LUETTIG B, DECKER T AND LOHMANN-MATTHES M-L. (1989). Evidence for the existence of two forms of membrane tumour necrosis factor: an integral protein and a molecule attached to its receptor. *J. Immunol.*, **143,** 4034–4038.

MCCARLEY DL, SHAH VO AND WEINER RS. (1983). Purified human monocyte subsets as effector cells in antibody-dependent cellular cytotoxicity. *J. Immunol.*, **131,** 1780–1783.

MASUCCI G, LINDEMALM C, FRODIN J-E, HAGSTROM B AND MELLSTEDT H. (1988). Effect of human blood mononuclear cell populations in antibody dependent cellular cytotoxicity (ADCC) using two murine (CO17-1A and Br55-2) and one chimeric (17-1A) monoclonal antibodies against a human colorectal cell line (SW948). *Hybridoma*, **7,** 429–440.

NATHAN C, BRUCKNER L, KAPLAN G, UNKELESS J AND COHN Z. (1980). The role of activated macrophages in antibody-dependent lysis of tumour cells. *J. Exp. Med.*, **152,** 183–197.

OKUSAWA S, GELFAND JA, IKEJIMA T, CONNOLLY RJ AND DINARELLO CA. (1988). Interleukin-1 induces a shock-like state in rabbits. *J. Clin. Invest.*, **81,** 1162–1172.

ONOZAKI K, MATSUSHIMA K, KLEINERMAN ES, SAITO J AND OPPENHEIM JJ. (1985*a*). Role of interleukin 1 in promoting human monocyte-mediated tumour cytotoxicity. *J. Immunol.*, **135,** 314–320.

ONOZAKI K, MATSUSHIMA K, AGGARWAL BB AND OPPENHEIM JJ. (1985*b*). Human interleukin 1 is a cytocidal factor for several tumor cell lines. *J. Immunol.*, **135,** 3962–3968.

ORTALDO J, WOODHOUSE C, MORGAN AC, HERBERMAN RB, CHERESH DA, PHILIP R AND EPSTEIN LB. (1987). Tumour necrosis factor as immunomodulator and mediator of monocyte cytotoxicity induced by itself, γ-interferon and interleukin-1. *Nature*, **323,** 86–88.

PULLYBLANK AM, GUILLOU PJ AND MONSON JRT. (1994). m17-1A, c17-1A and cSF25-mediated antibody-dependent cell-mediated cytotoxicity in patients with advanced cancer. *Br. J. Cancer*, **70,** 753–758.

QIAN J-H, TITUS JA, SANDREW SM, MEZZANZANICA D, GARRIDO MA, WUNDERLICH JR AND SEGAL DM. (1991). Human peripheral blood lymphocytes targeted with bispecific antibodies release cytokines that are essential for inhibiting tumor growth. *J. Immunol.*, **146,** 3250–3256.

RUGGERIO V AND BAGLIONI C. (1987). Synergistic antiproliferative activity of interleukin 1 and tumour necrosis factor. *J. Immunol.*, **138,** 661–663.

SMITH DM, LACKIDES GA AND EPSTEIN LB. (1990). Co-ordinated induction of autocrine tumour necrosis factor and interleukin 1 in normal human monocytes and the implications for monocyte-mediated cytotoxicity. *Cancer Res.*, **50,** 3146–3153.

STEPLEWSKI Z, HERLYN D, LUBECK M, KIMOTO Y, HERLYN M AND KOPROWSKI H. (1986). Mechanisms of tumor growth inhibition. *Hybridoma*, **5** (Suppl. 1), S59–S64.

STEPLEWSKI Z, LUBECK MD AND KOPROWSKI H. (1983). Human macrophages armed with murine immunoglobulin G2a antibodies to tumors destroy human cancer cells. *Science*, **221,** 865–867.

SUN LK, CURTIS P, RAKOWICZ-SZULCZYNSKA E, GHRAYEB J, CHANG N, MORRISON SL AND KOPROWSKI H. (1987). Chimeric antibody with human constant regions and mouse variable regions directed against carcinoma-associated antigen 17-1A. *Proc. Natl Acad. Sci. USA*, **84,** 214–218.

TAKAHASHI H, WILSON K, OZTURK M, MOTTE P, STRAUSS W, ISSELBACHER KJ AND WANDS JR. (1988). In vivo localization of human colon adenocarcinoma by monoclonal antibody binding to a highly expressed cell surface antigen. *Cancer Res.*, **48,** 6573–6579.

TAKAHASHI H, CARLSON R, OZTURK M, SUN S, MOTTE P, STRAUSS W, ISSELBACHER KJ, WANDS JR AND SHOUVAL D. (1989). Radioimmunolocation of hepatic and pulmonary metastasis of human colon adenocarcinoma (MAb SF-25). *Gastroenterology*, **96,** 1317–1329.

WEBB DSA, SHIMIZU Y, SEVENTER GAV, SHAW S AND GERRARD TL. (1990). LFA-3, CD44, and CD45: Physiologic triggers of human monocyte TNF and IL1 release. *Science*, **249,** 1295–1297.

WILSON KM, SIEGAL G AND LORD LM. (1989). Tumour necrosis factor-mediated cytotoxicity by tumour-associated macrophages. *Cell. Immunol.*, **123,** 158–165.